# The Reliability and Validity of the Polish Version of the Schema Mode Inventory (SMI)

**DOI:** 10.3390/jcm12196400

**Published:** 2023-10-08

**Authors:** Anna Grażka, Klara Królewiak, Klaudia Sójta, Dominik Strzelecki

**Affiliations:** 1 Department of Affective and Psychotic Disorders, Medical University of Lodz, 92-216 Lodz, Poland; annagrazka.psycholog@gmail.com (A.G.); klaudia.krakus@stud.umed.lodz.pl (K.S.); 2 Faculty of Psychology, SWPS University, 03-815 Warszawa, Poland; kkrolewiak@swps.edu.pl

**Keywords:** schema therapy, schema mode, psychometric properties, reliability, validity

## Abstract

(1) Background: Schema therapy and working with schema modes is increasingly popular. Since there is no validated tool to measure schema modes in Poland, in this study, we present an assessment of the psychometric properties of the Polish version the Short Schema Mode Inventory (SMI) adaptation. (2) Methods: First, the original version of the scale was translated. Subsequently, a validity and reliability study was conducted on a sample of 240 patients and 400 non-patients. In particular, the factor structure of the inventory was checked, internal reliability and intercorrelations between subscales were tested, differences between the clinical and non-clinical groups in terms of the severity of each factor were examined, and construct validity was assessed by comparing the association of results with external variables. (3) Results: The results did not conclusively confirm the 14-factor structure postulated in the original scale. Nevertheless, the relatively best fit indices were obtained for such a model. The internal reliability for the 14 subscales ranged from 0.74 to 0.95 (McDonald’s omega). Correlations between subscales demonstrated values from 0.03 to 0.72. The existence of differences between the clinical and non-clinical groups and the construct validity were confirmed. (4) Conclusions: The psychometric evaluation performed is mostly similar to the results obtained for other adaptations, and the results justify the inventory being used for research and clinical purposes when knowledge of its limitations is included.

## 1. Introduction

The concept of schema therapy, developed by Jeffrey E. Young [1], has been developing at a very fast pace over the past 30 years. There is an increasing amount of data on the effectiveness of interventions in different groups of patients, no longer only patients with borderline and narcissistic personality disorder (at whom this therapy was aimed at the beginning [2]), but also patients with other types of personality disorders [3] and different kinds of psychopathology such as depressive disorders, bipolar disorder, anxiety disorders, post-traumatic stress disorder, obsessive compulsive disorder, eating disorders, schizophrenia, and alcohol- and cocaine-dependence [4,5,6,7,8]. The interventions are effective in both individual and group work formats [9,10].

In the early stages of the development of schema therapy, a major role was attached to so-called early maladaptive schemas (EMSs), which arise in response to unmet early childhood needs. Young defined an EMS as: “a broad, pervasive theme or pattern, comprised of memories, emotions, cognitions, and bodily sensations, regarding oneself and one’s relationships with others, developed during childhood or adolescence, elaborated throughout one’s lifetime and dysfunctional to a significant degree” [2].

Over time, the second core construct of schema therapy, the so-called schema modes, began to receive more attention. Schema mode refers to “the dominant emotional state, coping patterns and reactions that are active for a person at a particular time” [11]. So, while EMSs are perceived as features, schema modes have been assigned the properties of states. In all likelihood, the focus on modes was motivated by their greater accessibility and clarity for clients, as well as the more intuitive possibilities of using them in experimental work, for example.

Young distinguished four groups of schema modes [2]. The first concerns the modes of the inner child, which are characterized by certain types of emotions experienced (sadness, feelings of helplessness, loneliness or shame, fear, and anger or rage that are difficult to control). These modes are revived when an individual finds himself in a situation where his emotional needs are not being met. The second group are parental modes, associated with a critical, blaming, or overly demanding voice, arising as a result of negative experiences in contact with significant others. The third group includes so-called coping modes, reflecting the overuse of three key belief coping strategies, namely compensation, avoidance, and confirmation. And the last category is the healthy modes, i.e., the Healthy Adult and Happy Child modes, through which an individual can lead a valuable, responsible, and at the same time joyful life. A more detailed description of the modes can be found in Appendix A (descriptions based on Lobbesteal et al. [12]). The objective of any therapeutic process is to take care of the inner child’s needs by removing parental modes, weakening coping modes, and strengthening healthy modes.

Also in the Polish context, in view of the growing number of studies indicating the effectiveness of interventions using schema modes, there is a noticeable focus on these constructs in seminars, or in therapeutic work with patients. Unfortunately, there is a lack of a measure with tested psychometric properties to evaluate the intensity of individual schema modes. This would support both the field of practice and allow for the conduct of research on modes in the Polish population. To date, several questionnaires have been developed to assess schema modes. The first was a questionnaire created by Young et al. [13], consisting of 270 items, which lacks an assessment of psychometric properties. Considering the large number of statements, Lobbesteal et al. [12] thought it would be a good solution to create a shortened version of it, which in its final form consisted of 118 statements. An adequate fit of the data to the 14-factor model, acceptable internal reliability of the tool, medium-to-high intercorrelations between subscales, and reasonable construct validity (in convergent and divergent aspects) were confirmed. Several adaptations of the aforementioned questionnaire have been developed so far, i.e., the German [14], Urdu [15], Greek [16], Danish [17], and Turkish versions [18]. Most have satisfactory psychometric properties for their use for both clinical and research purposes.

The purpose of this paper is to present the psychometric properties of the Polish adaptation. This was based on a shortened version of the Schema Mode Inventory (SMI) by Lobbesteal et al. [12]. An attempt was made to verify the reliability and validity of the measure, and therefore, in particular, the following were examined: factor structure, intercorrelations between the distinguished subscales, differences between the clinical and non-clinical groups and their strength, and validity in terms of convergent and divergent aspects.

## 2. Materials and Methods

### 2.1. Participants

The study included individuals between the ages of 18 and 70, without a diagnosis of intellectual disability, schizophrenia, or dementia syndromes, and who spoke Polish as a native speaker. A mixed sample was collected (*n* = 640), consisting of subjects from the clinical and general populations. Of these respondents, 36.1% (*n* = 231) declared that they had been given at least one of the following diagnoses in the course of their lives: depression, anxiety disorder, bipolar disorder, or alcohol dependence. A part of this group (i.e., 21.6%) declared a comorbid diagnosis of personality disorder. Of all respondents, 9.2% (*n* = 59) declared having a personality disorder diagnosis over the course of their lives. Meanwhile, 7.2% of respondents had experienced at least one psychiatric hospitalization in their life. Additionally, 17.2% received outpatient psychiatric care, 15.2% took psychiatric medications on a long-term basis, and 44.1% had had the experience of participating in psychotherapy. Due to lack of access to medical records, it was not possible to determine the exact characteristics of the study group in terms of current psychiatric diagnoses. A quarter of the respondents (25.8%) declared that they suffered from some somatic disease. 

Participants in the study were recruited over a period of about 15 months (from January 2020 to March 2021) within the departments of the Central Clinical Hospital in Lodz, the Mental Health Outpatient Clinic, and private practices run by doctors and psychotherapists. Participation in the study did not influence the design of the psychiatric care and pharmacological treatment undertaken. The remaining participants were selected amongst Medical University students and their surroundings by sending out an announcement to their email addresses. 

Due to the large number of statements for evaluation in connection with the construct validity examination, the entire study group was randomly divided into two subgroups (depending on the battery of tests being completed): Subgroup I (*n* = 311) and Subgroup II (*n* = 329). The demographic characteristics of the participants divided into groups are shown in Table 1.

### 2.2. Materials

#### 2.2.1. Schema Mode Inventory (SMI) 

This study involved an adaptation of the Dutch version of the Short Schema Mode Inventory [12], which was based on the Schema Mode Inventory, the long version constructed by Young et al. [13]. The Short SMI is a scale composed of 118 items assigned to 14 factors (described in more detail in Appendix A). Each factor contains between 4 and 10 items. The questionnaire is preceded by instructions asking respondents to rate each statement in terms of how often they believe or feel the way described. The individual items are rated on a 6-point Likert scale, where 1 is “never or almost never” and 6 is “all of the time”. The results are obtained by calculating the arithmetic mean for each subscale. There is no cut-off point. The higher the average score obtained in the subscale, the greater the severity of the tested factor.

The results of the confirmatory factor analysis proved that the original version of the Short Schema Mode Inventory (by Lobbesteal et al. [12]) is characterized by good levels of the goodness-of-fit indices of the model to the data using the 14-factor solution. The following results were obtained: comparative fix index (cFI) = 0.98, non-normed fit index (NNFI) = 0.98, standardized root mean square (SRMR) = 0.066, root mean square error of approximation (RMSEA) = 0.053, and chi-square test (χ^2^) (degrees of freedom, df) = 18,374.70 (6694). Mean item loadings per subscale varied between 0.53 and 0.85. The internal consistency of the subscales was acceptable (Cronbach’s α ranged from 0.79 to 0.96). Positive correlations were noted between all maladaptive modes and between the two adaptive modes. The maladaptive modes correlated negatively with the adaptive ones [12]. 

The procedure for adaptation of the inventory described above was preceded by obtaining written permission from the authors of the original short version. The adaptation was made using the method of translation, that is, by means of a fairly faithful translation, in which minor modifications are allowed in places where a literal translation would not be possible [19]. 

As a first step, in accordance with the adopted standards, the original version of the Short SMI was translated into Polish by two independent psychologists fluent in English with theoretical and practical knowledge of schema therapy. A comparison of the translation results revealed that the discrepancies found in 8 items (i.e., 1, 18, 50, 52, 70, 79, 116, 118) were mainly due to the use of synonymous words or due to the difficulty in maintaining translation accuracy and simultaneous semantic equivalence of statements. Through a group discussion on the selection of the most optimal versions, the wording of most items was established. A native Polish speaker with a background in psychology, who has lived in the United States for years, was then asked to perform a blinded back-translation. Any additional translation discrepancies that arose were resolved through discussion among the initial translators.

Finally, a pilot study was performed with the participation of 34 students, who were able to make comments on the design of the questionnaire and their understanding of the various statements. The result was a change in the wording of two items. The average time to complete the questionnaire was 25 min. This validated the official Polish language version of the questionnaire, which was then subjected to psychometric evaluation.

The characteristics of the other measures used in the assessment of criterion validity are presented below.

#### 2.2.2. Temperament and Character Inventory (TCI, by Cloninger at al. [20])

The TCI is a questionnaire based on the psychobiological model developed by Cloninger [20]. It contains scales to measure various dimensions of temperament and character. It is composed of 240 statements, to which the respondent refers by choosing one of two options: true or false. This study used the Polish version of this scale adapted by Hornowska [21], which has psychometric properties that enable it to be used for research purposes. The scales applied have a reliability (measured by the Kuder–Richardson reliability coefficients, KR_20_) ranging from 0.42 to 0.87, with a mean of 0.67. In the current group, internal consistency for the compared subscales (Cronbach’s α calculated for dichotomous data) ranged from 0.46 to 0.84 with a mean of 0.68. 

The inventory distinguishes seven scales, within which 25 subscales are identified. Sixteen of them were used to test construct validity in this study. Positive correlations are assumed to occur between the TCI fear of uncertainty scale and the Vulnerable Child mode; the TCI impulsiveness scale and the Impulsive Child mode; the TCI persistence scale and the Undisciplined Child mode; the TCI dependence mode and the Compliant Surrender mode; the TCI persistence scale and the Demanding Parent mode; and all the TCI self-directedness subscales and all the cooperativeness subscales and the Healthy Adult mode. Negative correlations were expected between the TCI responsibility scale and the Impulsive Child mode; the TCI purposeful scale and the Undisciplined Child mode; the TCI resourcefulness and the Vulnerable Child and Compliant Surrender modes; the TCI enlightened second nature scale and the Detached Self-Soother, Undisciplined Child, and Impulsive Child modes; all the TCI cooperativeness subscales and the Angry Child mode; the TCI compassion scale and the Enraged Child and Self-Aggrandizer modes; all the TCI self-directedness subscales, the TCI attachment scale and the Detached Protector mode; and the TCI compassion scale and the Bully and Attack mode.

#### 2.2.3. Coping Inventory for Stressful Situations (CISS, by Endler and Parker [22])

The CISS is a questionnaire developed based on the interactional theoretical model. It measures stress coping styles, understood as conscious coping actions that an individual takes when faced with a specific stressful situation. These actions are the result of the interaction that occurs between the characteristics of the situation and the individual’s characteristic style. The scale allows the assessment of three dimensions: task-oriented style, emotion-oriented style, and avoidance-oriented style, where there are two subscales: distraction and social diversion [23].

The questionnaire is composed of 48 statements describing various behaviors undertaken in a stressful situation. Respondents rate each statement on a 5-point Likert scale, indicating the frequency with which they engage in a given action, where 1 means “never” and 5 means “very often”. The current study used a Polish adaptation of the questionnaire, developed by Szczepaniak et al. [24]. This scale is characterized by adequate psychometric properties, i.e., internal consistency with Cronbach α ranged from 0.6 to 0.88 (with a mean of 0.77) and satisfactory construct validity. In the current study, reliability indices were in the range of 0.74 to 0.90 (with a mean of 0.82). Positive correlations are predicted between the CISS task-oriented style and the Healthy Adult mode; CISS emotional-oriented scale and the Vulnerable Child mode; and the CISS distraction scale and the Detached Self-Soother mode; negative correlations are predicted between CISS social diversion and the Detached Protector mode.

#### 2.2.4. Rosenberg Self-Esteem Scale (SES [25])

The SES is a tool that measures self-esteem, that is, positive or negative attitudes toward the Self [25]. The scale consists of 10 statements that respondents address on a 4-point scale from “strongly agree” to “strongly disagree”. The current study used a Polish adaptation of the measure, developed by Dzwonkowska et al. [26]. The adapted questionnaire’s internal consistency ranged between 0.81 and 0.83 for Cronbach’s α for the whole scale, depending on the study sample. In the original version, the single-factor structure of the measure was proven. The Polish version assumes the existence of two factors (one composed of positive statements, the other of negative ones). Cronbach’s α in the current sample was at the level of 0.90. A positive relationship is expected between the SES score and the Healthy Adult mode, a negative one between the SES score and the Punitive Parent mode.

#### 2.2.5. State–Trait Anger Expression Inventory-2 (STAXI-2 [27])

The STAXI-2 is a revised version of the STAXI questionnaire developed by Spielberger et al. [27], which measures anger in various aspects. The scale contains 57 statements, which respondents rate on a 4-point Likert scale from “definitely no” to “definitely yes”. The questionnaire is divided into three parts: one measuring anger as a state, one measuring anger as a trait, and one dealing with expressing and controlling anger. This study used the scale in an adaptation developed by Bąk [28]. Internal consistency coefficients (Cronbach’s α) for the Polish adaptation range from 0.74 to 0.95 for each of the subscales. The validity of the measure and its relative stability in terms of anger as a trait were also confirmed. The current sample obtained good-to-excellent reliability indices: for the anger-as-state scale 0.95, for the anger-as-trait scale 0.86, for the other subscales 0.73–0.93.

Referring to the perception of schema modes in the category of states [11], positive correlations are predicted to occur between the subscales of the STAXI-2 considering anger as a state and the Angry Child and Enraged Child modes; between the STAXI-2 anger expression-out scale and the Angry Child, Enraged Child, and Bully and Attack modes; the STAXI-2 anger expression-in scale and the Vulnerable Child and Compliant Surrender modes; and between the STAXI-2 anger control-out and anger control-in scales and the Healthy Adult mode. Negative correlations are expected between anger control-out scale and the Angry Child, Enraged Child and Impulsive Child modes and the anger control-in scale and the same three child modes.

### 2.3. Procedure

The study was approved by the Bioethics Committee of the Medical University of Lodz (RNN/302/19/KE, 9 July 2019). The data used in the study were obtained partly by face-to-face contact (paper-and-pencil method)—321 records (46.7% of the clinical group and 52.3% of the non-clinical group)—and partly via an online survey—319 records (53.3% of the clinical group and 47.3% of the non-clinical group). The decision to include an online survey group was made in the context of the COVID-19 pandemic, which resulted in a problem with access to volunteers for the study. 

Each participant was tested individually. The randomly selected subgroups were given batteries composed of various questionnaires. Subgroup I completed a questionnaire that was the Polish translation of the Short Schema Mode Inventory (SMI, Version 1) and the Temperament and Character Inventory (TCI). Subgroup II, on the other hand, filled out a battery that also consisted of a Polish translation of the Short SMI and three other questionnaires, i.e., the Coping Inventory for Stressful Situations (CISS), Rosenberg Self-Esteem Scale (SES), and State–Trait Anger Expression Inventory-2 (STAXI-2). This procedure significantly reduced the time needed to fill out the battery (in the end, the average battery filling time was about 45 min) in order to keep the subjects engaged and reduce the impact on results of the fatigue factor.

Participants were informed in the instructions preceding the set of questionnaires about the identity of the researcher and the purpose of the study and its procedure. They were also assured of the scientific nature of the study, their voluntary participation, and the complete anonymity of the data provided in the questionnaires. To increase the motivation to respond honestly to the evaluated items, willing participants could receive a brief psychological characterization based on the results achieved.

### 2.4. Statistical Analyses 

To perform the analyses, the following statistical techniques were used: confirmatory factor analyses (cFA) for assessing the factor structure of the adapted questionnaire, the McDonald’s omega coefficient for estimating internal consistency reliability for the subscales, independent sample *t*-test to determine differences between the means, and Spearman correlation coefficient with Bonferroni correction to establish intercorrelation between SMI subscales and correlations of the SMI subscales and other analyzed variables. For the statistical calculations, the statistical package PS IMAGO PRO 8.0 and the AMOS 8.0 were used.

## 3. Results

### 3.1. Factor Structure of the Polish Version of the Short SMI

The present study tried to reflect the factor structure of the original version of the scale, so it was verified using CFA. A maximum likelihood estimator, resistant to not fulfilling the assumption of normality of the distribution of the variable under study, was applied. According to the standard [29], several measures of the model’s fit to the data were used: corrected chi-square (χ^2^), chi-square divided by the number of degrees of freedom (χ^2^/*df*), comparative fix index (CFI), non-normed fit index (NNFI), standardized root mean square residual (SRMR), and root mean square error of approximation (RMSEA). The following threshold values were adopted: *p*-value for the model > 0.05, χ^2^/*df* < 3, CFI ≥ 0.90, NNFI ≥ 0.95, SRMR < 0.08, and RMSEA < 0.08 [30]. 

A 14-factor structure was compared with an 8-factor and 4-factor model (all based on theoretical assumptions). Subsequent models were created taking into account correlations between latent variables. Given the differences found in the frequency of distributions of the items, Tabachnick and Fidell [31] suggest that a loading value of 0.32 can be used as a cut-off point. Accordingly, six items were removed (two from the Bully and Attack factor, two from the Happy Child factor, and one each from the Healthy Adult and Self-Aggrandizer mode). In the next step, covariances were added between error terms within the same factor, where Modification Indices ≥ 20 [32]. Table 2 presents the goodness-of-fit indices for the proposed models.

According to the results shown in Table 2, the Polish version does not adequately fit the proposed 14-factor factor model because, although the normed χ^2^ and RMSEA give adequate fit values, in the other indices of the confirmatory factor analysis (CFI, NNFI, and SRMR), adequate values are not obtained. The relative best was the results obtained for the 14-factor model, consisting of 112 items. It could be said that the fit indices are valid. Based on these data, it can be confirmed that this model presents an absolute fit, but not an incremental fit. The parsimony fit is not known, as the AIC index was not obtained. Unquestionably, the issue of the factor structure of the Polish version of the inventory requires further analysis.

### 3.2. Internal Consistency, Item Loadings, and Correlations between Subscales of the Short SMI

The reliability analysis revealed that the internal consistency values for the specific subscales of the Polish version of the Short SMI (see Table 3) ranged from acceptable (lowest ω = 0.74 for Bully and Attack mode) to excellent (ω = 0.95 for Vulnerable Child mode) with a mean of ω = 0.84 (referring to the generally accepted cut-off point of 0.7 [33]), which is similar to the result obtained in the original version [12] and other adaptations [14,16,17,18]. It is noteworthy that in most analyses, one of the less reliable scales appears to be the Bully and Attack mode [14,15,17,18].

According to the criterion adopted following Tabachnick and Fidell [31], all factor loadings for items have assumed adequate values (see Table 4). The mean factor loadings for each subscale for the 14-factor solution ranged from 0.54 to 0.79, with an overall mean of 0.64 (see Table 3).

The mean item correlations within each factor ranged between 0.31 and 0.63 (with a mean correlation of 0.43; see Table 3). The factor intercorrelations between subscales are summarized in Table 5. Positive correlations were proven between both the maladaptive modes and the two adaptive modes. The adaptive modes correlated negatively with the maladaptive modes. The only exception was four correlation values, which proved to be non-significant. The mean correlation between all maladaptive child modes (VC, AC, EC, IC, and UC) was 0.49, the mean correlation between coping modes (CS, DPT, DSS, SA, and BA) was 0.30 (including correlation between compensation modes, i.e., SA and BA, at 0.54, and between avoidance-based modes, i.e., DPT and DSS, at 0.44), parent modes (PP and DP) correlate with a factor of 0.52 with each other, and healthy modes (HA and HC) correlate with a factor of 0.71 with each other. In some cases, high correlations were obtained, but despite this, none of the confidence intervals (±2 × SE, [34]) around the estimated correlations between the two subscales included 1.0. This suggests that the subscales of the adapted version are distinct constructs.

### 3.3. Differences and Effect Size between Subgroups

As part of the assessment of the construct validity of the scale, it was checked whether there were differences between the mean of the subscales for the clinical (*n* = 240) and non-clinical group (*n* = 400). The clinical group consisted of people who had experienced a psychiatric diagnosis that included disorders such as depression, anxiety disorder, bipolar disorder, schizophrenia, or alcohol dependence at least once in their lives, and people with a diagnosis of personality disorder (including those where both categories of disorders co-occurred). This solution was decided because nine people were identified in the study group with a diagnosis of personality disorder only, which presented too small a group for comparison.

The results show that there are significant differences between the clinical and non-clinical groups, with the exception of the Bully and Attack and Self-Aggrandizer modes (at *p*-value ≤ 0.05; see Table 6). Within the adaptive modes, significantly higher scores were obtained in the clinical group compared with the non-clinical group. Under adaptive modes, the result was the opposite, i.e., the scores in the clinical group were significantly lower compared with the scores in the non-clinical group. The most significant difference between the groups was observed between the intensity of the Happy Child mode (Cohen’s δ = −0.57), while the lowest was between the Demanding Parent mode scores (Cohen’s δ = 0.16).

### 3.4. Criterion Validity

Table 7 and Table 8 show the correlation coefficients of assumed and not assumed relationships between subscales of the Polish version of the Short SMI and theoretically related external constructs. All hypothesized correlations turned out to be significant. Most of them took moderate to strong values (Spearman’s ρ value of 0.3–0.5 and 0.5–0.7, respectively), with the exception of the correlations between the Healthy Adult mode and all the TCI cooperativeness subscales (i.e., social acceptance, empathy, helpfulness, compassion, pure-hearted conscience). In addition to the assumed relationships, a number of unanticipated correlations were also revealed, but mostly of lower strength. Moreover, 144 of 305 (47.2%) correlations took a value below 0.3, indicating good discriminant validity.

Analyzing the associations of schema modes with measures of temperament and character, it is observed that there are positive correlations with adaptive modes (i.e., the Healthy Adult and Happy Child modes) and negative correlations with non-adaptive modes. The exception is the TCI impulsivity scale, which is positively correlated with the child maladaptive scales. Noteworthy are the reasonably high negative correlations with coping modes (i.e., the Compliant Surrender, Detached Protector, Self-Aggrandizer, and Bully and Attack modes), which indicate good discriminant validity.

The predicted correlations obtained with the CISS subscales proved to be among the highest, although several unpredicted ones also took high values. A number of correlations with strengths above 0.5 were found with the CISS emotion-oriented scale (positive with the Angry Child, Compliant Surrender, Detached Self-Soother, Punitive Parent, and Demanding Parent modes and negative with the Happy Child and Healthy Adult modes). The CISS avoidant-oriented style scale showed weak associations, and only its subscales (i.e., distraction and social diversion) allowed for stronger correlations with the schema modes.

In addition to the predicted correlations, unanticipated relationships of high strength were also reported between SES score and schema modes. With the exception of the Self-Aggrandizer mode, all others significantly correlated with the self-esteem dimension. The strongest negative correlations (ρ > 0.5) were obtained between SES score and the Vulnerable Child, Compliant Surrender, Detached Protector, and Punitive Parent modes. Very strong positive relationships (ρ > 0.7) were achieved between the SES score and the Healthy Adult and Happy Child modes.

Also noteworthy are the correlations obtained between STAXI-2 subscales and schema modes by the SMI. Contrary to predictions, the schema modes associated with anger regulation difficulties (i.e., the Angry Child and Enraged Child modes) turned out to correlate more strongly with STAXI-2 subscales treating anger as a trait rather than a state. High correlations of this scale were also obtained with compensatory coping modes (i.e., the Self-Aggrandizer and Bully and Attack modes). A fairly high discriminant value was presented by the scales of anger expression-out and -in and the scales of anger control-out and -in relative to child modes associated with anger (i.e., the Angry Child and Enraged Child modes). Not entirely as expected, the scale of anger control-out and -in showed only a moderate strength of association with the Healthy Adult mode. 

In conclusion, 21 out of 49 predicted correlations had a value of at least 0.5 in the current sample. Additionally, several other significant correlations were found, which are theoretically convergent with the SMI subscales. For most of the subscales (with the exception of the Demanding Parent mode), correlations at the level of at least a strong relationship were obtained. In addition, the moderate discriminatory validity of the adapted inventory was confirmed.

## 4. Discussion

This study describes the procedure for adaptation of the Short Schema Mode Inventory (SMI) to Polish conditions and verifies the psychometric properties of the obtained version. The need to adapt the questionnaire was based on the growing popularity of the application of schema modes in schema therapy, both in the area of scientific research and in the everyday clinical practice of psychotherapists. Evidence of the efficacy of using schema modes in the treatment of mental disorders is increasing, which implies the need for evaluation of their reliability, also in the Polish context.

The results of the analyses obtained are mostly similar in strengths and weaknesses to both the psychometric properties of the original Dutch version of the SMI [12] and other language versions [14,15,16,17,18]. The study proved that the most promising direction for consideration is the 14-factor solution. An acceptable reliability of the measure’s individual subscales, moderate-to-high correlations between subscales, and satisfactory results within the context of criterion validity were confirmed when testing intergroup differences and the convergent as well as divergent aspect of validity.

Examining the conducted factor analysis in more detail, it should be emphasized that the results obtained are not unambiguous. Admittedly, the best values of fit indices were obtained with a 14-factor model, consisting of 112 items, taking into account correlations between factors and covariances between error terms, but even with this model, only some of the factors indicated a good fit (i.e., the normed χ^2^ and RMSEA). With this data, we obtained an absolute fit, but not an incremental fit. Similar results were obtained in a Danish study [17]. The authors of this study pointed out that confirmatory factor analysis is too restrictive, resulting in an overestimation of the correlations between latent variables. As a solution, they proposed using exploratory structural equation modeling (ESEM), which more adequately estimates these correlations. However, this would contradict the methodology of the original questionnaire adaptation, where using CFA yielded appropriate fit indices. In all likelihood, therefore, the cause lies somewhere else.

Apart from the possibility of mistakes made at the semantic translation level, another explanation could be cultural differences between the populations of the countries where the questionnaire was adapted. It would be valuable to compare the distributions of results in various populations. Moreover, the present study reflects the distribution of results in an age-heterogeneous group. It would be worthwhile to investigate, more specifically, the age dependence of the severity of individual schema modes. Due to the small percentage of people over 50 years old in the present study (i.e., 9.4%), we refrained from considering age as a covariate variable. Nevertheless, there are several studies that show that personality traits undergo modifications with age (cf. [35]). We can expect a similar phenomenon regarding the severity of dysfunctional schema modes, which would require empirical verification. 

Also noteworthy is the proportion of people surveyed. In the original study, the clinical population accounted for about 53.8% of the subjects, of which about two-thirds were patients with a diagnosis of personality disorder, thus better differentiating the distribution in terms of mode severity. In the group analyzed in this study, the clinical population comprised 37.5%, and the group of subjects with a diagnosis of personality disorder was so small and heterogeneous that it was not singled out for separate analysis. A limitation of our survey is undoubtedly also the way we assessed the presence/absence of mental or personality disorders. Respondents self-reported the diagnoses given to them so far, which may be a source of bias in the results. Future studies should take care to implement external evaluation measures.

Perhaps we can partly explain the results obtained by the predominance of women in the study group. There is a lack of studies considering schema modes for gender differences. However, there are a few reports showing that women differ from men regarding the occurrence of early maladaptive schemas (e.g., [36,37,38]), which implies the conclusion that mode differences are also possible. This issue requires further verification.

Another explanation for this configuration of results could be the fact that the method used (i.e., maximal likelihood) was not robust enough to not follow the assumption of normality of distribution. In such situations, the use of the asymptotically distribution-free (ADF) method is postulated [39], which significantly increases the required number of tested observations (in this study to about 7000). Due to the insufficient number of observed variables, this method could not be tested. Further limitations may have followed from the high sensitivity of the χ^2^ statistic to the size of the study group and to the complexity of the model [40], which with 118 items is unavoidable. Therefore, we relied on the normalized value of the statistical χ^2^. Similar properties characterize the CFI and NNFI indices, which, while quite robust to sample size, show strong sensitivity regarding a high model complexity [39]. In light of the above, it would be valuable to conduct a factor analysis using a more homogeneous group, possibly using a different evaluation method.

The question of applying factor analysis itself to data that were intended to represent states also requires consideration. Nezlek argues that while trait structure can be modeled using a factor approach based on single-level data, modeling state variability requires multiple measurements, for which a multilevel analysis is appropriate [41]. It would therefore be extremely valuable to design a study that would include multiple measurements of schema modes in situations that differ, for example, in the level of emotional arousal.

Taking the above issues into consideration, we decided to stay with the questionnaire’s original factor structure for the purposes of further current analyses. This is due to the fact that—regardless of questionable fit indices—the internal consistency of the particular subscales was at least moderate to high. An additional argument is the facade validity of the measure, which is justified by the use of factor division in clinical practice. Reducing the number of dimensions would result in a loss of nuances of meaning, determining the patient’s presentation of a particular mode.

To highlight the factor structure, the mean factor loadings for the subscales were checked, and also take acceptable-to-high scores. Following Tabachnick and Fidell’s criterion [31], a fairly attenuated cut-off point, i.e., 0.32, for individual items was adopted. This procedure was guided by an attempt to adapt the measure as closely as possible to the original. A more restrictive cut-off point for factor loadings would have required the removal of more items, which would have affected the structure of the inventory, making comparisons more difficult in perspective. At the same time, we suggest that future research consider conducting an exploratory factor analysis to see what factors the Polish population presents in this questionnaire. It would be recommended to aim at the original version of the questionnaire by Young, consisting of 270 items, which would facilitate possible item reduction dictated, for example, by semantic differences.

In subsequent analyses, we examined construct validity. Mostly moderate correlations were obtained between subscales: positive between adaptive, and positive between maladaptive and negative between the two groups. These results indicate the co-occurrence of adaptive modes and the co-occurrence of maladaptive modes. At the same time, the moderate values of the correlation coefficients show that we are referring to distinct constructs.

The study also proved the existence of significant differences between the scores obtained in the clinical group compared with the non-clinical group. Individuals in the clinical group scored significantly higher on maladaptive modes and lower on adaptive modes compared with the non-clinical group. The results collected are consistent with Young et al.’s theoretical assumptions [13] and the data in the adaptations of the questionnaire that have been developed so far [12,14,16,17,18].

Only the Bully and Attack and Self-Aggrandizer modes did not differentiate between the group with psychiatric diagnoses and the healthy group. Perhaps this is a consequence of the fact that these are the scales with the lowest mean factor loadings. A second explanation may be related to the hypothesis that these are modes that differentiate more between individuals with a personality disorder diagnosis while not suffering from other types of mental disorders. These suppositions require further verification.

It is noteworthy that the greatest strength of the effect of comparisons between groups characterized the Happy Child and Vulnerable Child modes. It is observed that in clinical practice, a very significant role is attached to working with the Vulnerable Child mode, so that the patient is able to meet his needs. At the same time, it is only recently that attention has begun to be paid to the Happy Child mode, which, according to the data obtained, is an extremely important predictor of mental health.

A further comparison was made using a mixed clinical group consisting of subjects suffering from different types of mental disorders (such as depression, anxiety disorder, bipolar disorder, schizophrenia, or alcohol dependence) and personality disorders. The severity of the disorders at the time of the study was not controlled. It certainly would be valuable to see whether the Polish version of the questionnaire also differentiates the group of patients with so-called Axis I versus those with personality disorders (Axis II, as, for example, in the study by Lobbesteal et al. [12]). It also would be recommended to control for the severity of the disorder at the time of testing.

The final step in testing criterion validity was to examine the associations of schema modes with external constructs (i.e., measures of temperament and character, coping styles, self-esteem, and anger in terms of trait and state). All predicted correlations proved significant. Most of them took at least a moderate value. There were also many unpredicted significant correlations that showed lower values compared with the hypotheses. It can be concluded that the obtained data indicate satisfactory theoretical validity in both convergent and divergent aspects. 

An interesting result, contrary to prediction, was the higher correlations obtained between anger in the sense of a trait rather than a state. It opens the field for expanding the analysis to include the induction of affect and its importance in “triggering” modes.

In addition to the indicated modifications and new research directions, the literature and clinical practice acknowledge the need to distinguish new modes. Already in the original version of the questionnaire, the Over Controller or the Lonely Child and Abandoned and Abused Child modes were distinguished [13]. Recent research abounds with newer modes (including for example: the Helpless Surrender, Pollyanna, or Paranoid Controller modes; cf. [42]). It is possible that the inclusion of more scales will be a further direction in the development of the adapted tool, though certainly this should be done with consideration given to their discriminative value and the utility of the measure for research and clinical purposes.

In conclusion, the results provide information on the psychometric properties of the Polish version of the Short SMI and further directions for needed research. The measure’s application to clinical and research purposes should be conducted with the awareness of its described limitations. Next to a professional clinical diagnosis, the scale can provide significant support in psychotherapeutic work, indicating the schema modes most often used by the respondent (both in the diagnostic layer and in the context of assessing effectiveness of treatment). In this area of research, the adapted inventory will allow us to compare the results obtained in Poland with results from other countries and facilitate the development of schema therapy in accordance with the principles of evidence-based medicine.

## Figures and Tables

**Table 1:** Characteristics of the study sample.

Demographic Characteristics	Whole Sample	Subgroup I	Subgroup II	Missing Data
*n*	640	311	329	
Gender, *n* (%):				*n* = 8
Female	545 (85.2%)	261 (84.7%)	284 (87.7%)	
Male	87 (13.6%)	47 (15.3%)	40 (12.3%)	
Age; mean (SD)	34.04 (10.62)	34.76 (10.73)	33.35 (10.45)	
Education; *n* (%)				*n* = 2
higher	347 (54.2%)	185 (59.9%)	162 (49.2%)	
secondary	269 (42.0%)	110 (35.6%)	159 (48.3%)	
basic vocational	14 (2.2%)	9 (2.9%)	5 (1.5%)	
elementary	8 (1.3%)	5 (1.6%)	3 (0.9%)	
Place of residence				*n* = 1
village	123 (19.2%)	53 (17.0%)	70 (21.3%)	
city under 100,000 inhabitants	177 (27.7%)	76 (24.4%)	101 (30.8%)	
city over 100,000 inhabitants	339 (53.0%)	182 (58.8%)	157 (47.9%)	
Marital status				*n* = 5
married	186 (29.1%)	96 (31.3%)	90 (27.4%)	
in an informal relationship	200 (31.3%)	95 (30.9%)	105 (32.0%)	
single	249 (38.9%)	116 (37.8%)	133 (40.5%)	
Employment				*n* = 2
employed/student	557 (87%)	273 (88.1%)	284 (86.6%)	
retired	13 (2.0%)	6 (1.9%)	7 (2.1%)	
unemployed	68 (10.6%)	31 (10.0%)	37 (11.3%)	

**Table 2:** Goodness-of-fit indices of various tested models (*n* = 640).

Model	χ^2^ (df)	Normed χ^2^	CFI	NNFI	SRMR	RMSEA
14 correlated factors (118 items)	19,426.263 (6694) ***	2.902	0.715	0.624	0.122	0.055
14 correlated factors with covariances (118 items)	16,798.122 (6640) ***	2.530	0.773	0.674	0.118	0.049
14 correlated factors with covariances (112 items)	15,064.511 (5960) ***	2.528	0.790	0.697	0.112	0.049
8 correlated factors (118 items)	22,626.77 (6757) ***	3.349	0.645	0.561	0.137	0.061
8 correlated factors with covariances (118 items)	17,024.863 (6618) ***	2.573	0.767	0.670	0.128	0.050
4 correlated factors (118 items)	28,516.757 (6779) ***	4.207	0.514	0.447	0.142	0.071

Note: χ^2^ = chi-square; df = degrees of freedom; CFI = comparative fix index; NNFI = non-normed fit index; SRMR = standardized root mean square residual; RMSEA = root mean square error of approximation; *** *p* < 0.001.

**Table 3:** Internal reliability of the Polish version of the Short SMI (*n* = 640).

SMI Subscales	Number of Items	Mean Inter-Item Correlation	McDonald’s Omega	Mean Item Loading
Vulnerable Child	10	0.63	0.95	0.79
Angry Child	10	0.34	0.83	0.57
Enraged Child	9	0.43	0.88	0.63
Impulsive Child	8	0.44	0.87	0.65
Undisciplined Child	5	0.46	0.81	0.67
Happy Child	8	0.58	0.92	0.75
Compliant Surrender	7	0.39	0.82	0.63
Detached Protector	9	0.46	0.89	0.67
Detached Self-Soother	4	0.47	0.79	0.69
Self-Aggrandizer	9	0.31	0.80	0.54
Bully and Attack	7	0.31	0.74	0.55
Punitive Parent	10	0.48	0.90	0.69
Demanding Parent	7	0.34	0.79	0.57
Healthy Adult	9	0.35	0.83	0.59
Mean	8	0.43	0.84	0.64

**Table 4:** Item loadings for 14-factor model (*n* = 640).

	SMI Item	Scale	Item Loading
1.	I demand respect by not letting other people push me around.	BA	0.01
2.	I feel loved and accepted.	HC	0.75
3.	I deny myself pleasure because I don’t deserve it.	PP	0.66
4.	I feel fundamentally inadequate, flawed, or defective.	VC	0.78
5.	I have impulses to punish myself by hurting myself (e.g., cutting myself).	PP	0.59
6.	I feel lost.	VC	0.79
7.	I’m hard on myself.	DP	0.66
8.	I try very hard to please other people in order to avoid conflict, confrontation, or rejection.	CS	0.68
9.	I can’t forgive myself.	PP	0.74
10.	I do things to make myself the center of attention.	SA	0.48
11.	I get irritated when people don’t do what I ask them to do.	SA	0.59
12.	I have trouble controlling my impulses.	IC	0.79
13.	If I can’t reach a goal, I become easily frustrated and give up.	UC	0.69
14.	I have rage outbursts.	EC	0.77
15.	I act impulsively or express emotions that get me into trouble or hurt other people.	IC	0.81
16.	It’s my fault when something bad happens.	PP	0.66
17.	I feel content and at ease.	HC	0.85
18.	I change myself depending on the people I’m with, so they’ll like me or approve of me.	CS	0.47
19.	I feel connected to other people.	HC	0.52
20.	When there are problems, I try hard to solve them myself.	HA	0.12
21.	I don’t discipline myself to complete routine or boring tasks.	UC	0.71
22.	If I don’t fight, I will be abused or ignored.	AC	0.57
23.	If you let other people mock or bully you, you’re a loser.	BA	0.42
24.	I physically attack people when I’m angry at them.	EC	0.41
25.	Once I start to feel angry, I often don’t control it and lose my temper.	EC	0.84
26.	It’s important for me to be Number One (e.g., the most popular, most successful, most wealthy, most powerful).	SA	0.65
27.	I feel indifferent about most things.	DPT	0.56
28.	I can solve problems rationally without letting my emotions overwhelm me.	HA	0.65
29.	I won’t settle for second best.	SA	0.51
30.	Attacking is the best defense.	BA	0.54
31.	I feel cold and heartless toward other people.	DPT	0.51
32.	I feel detached (no contact with myself, my emotions or other people).	DPT	0.75
33.	I blindly follow my emotions.	IC	0.62
34.	I feel desperate.	VC	0.81
35.	I allow other people to criticize me or put me down.	CS	0.72
36.	In relationships, I let the other person have the upper hand.	CS	0.68
37.	I feel distant from other people.	DPT	0.79
38.	I don’t think about what I say, and it gets me into trouble or hurts other people.	IC	0.65
39.	I work or play sports intensively so that I don’t have to think about upsetting things.	DSS	0.53
40.	I’m angry that people are trying to take away my freedom or independence.	AC	0.40
41.	I feel nothing.	DPT	0.75
42.	I do what I want to do, regardless of other people’s needs and feelings.	SA	0.37
43.	I don’t let myself relax or have fun until I’ve finished everything I’m supposed to do.	DP	0.38
44.	I throw things around when I’m angry.	EC	0.55
45.	I feel enraged toward other people.	AC	0.69
46.	I feel that I fit in with other people.	HC	0.61
47.	I have a lot of anger built up inside of me that I need to let out.	AC	0.68
48.	I feel lonely.	VC	0.78
49.	I like doing something exciting or soothing to avoid my feelings (e.g., working, gambling, eating, shopping, sexual activities, watching TV).	DSS	0.72
50.	Equality doesn’t exist, so it’s better to be superior to other people.	BA	0.63
51.	When I’m angry, I often lose control and threaten other people.	EC	0.54
52.	I let other people get their own way instead of expressing my own needs.	CS	0.82
53.	If someone is not with me, he or she is against me.	AC	0.61
54.	In order to be bothered less by my annoying thoughts or feelings, I make sure that I’m always busy.	DSS	0.80
55.	I’m a bad person if I get angry at other people.	PP	0.57
56.	I don’t want to get involved with people.	DPT	0.53
57.	I feel that I have plenty of stability and security in my life.	HC	0.76
58.	I know when to express my emotions and when not to.	HA	0.56
59.	I’m angry with someone for leaving me alone or abandoning me.	AC	0.51
60.	I don’t feel connected to other people.	DPT	0.66
61.	I can’t bring myself to do things that I find unpleasant, even if I know it’s for my own good.	UC	0.68
62.	I break rules and regret it later.	IC	0.43
63.	I feel humiliated.	VC	0.70
64.	I trust most other people.	HC	0.31
65.	I act first and think later.	IC	0.55
66.	I get bored easily and lose interest in things.	UC	0.66
67.	Even if there are people around me, I feel lonely.	VC	0.80
68.	I don’t allow myself to do pleasurable things that other people do because I’m bad.	PP	0.72
69.	I assert what I need without going overboard.	HA	0.41
70.	I feel special and better than most other people.	SA	0.52
71.	I don’t care about anything; nothing matters to me.	DPT	0.73
72.	It makes me angry when someone tells me how I should feel or behave.	AC	0.43
73.	If you don’t dominate other people, they will dominate you.	BA	0.62
74.	I say what I feel, or do things impulsively, without thinking of the consequences.	IC	0.66
75.	I feel like telling people off for the way they have treated me.	AC	0.70
76.	I’m capable of taking care of myself.	HA	0.68
77.	I’m quite critical of other people.	SA	0.61
78.	I’m under constant pressure to achieve and get things done.	DP	0.66
79.	I’m trying not to make mistakes; otherwise, I’ll get down on myself.	DP	0.75
80.	I deserve to be punished.	PP	0.81
81.	I can learn, grow, and change.	HA	0.67
82.	I want to distract myself from upsetting thoughts and feelings.	DSS	0.72
83.	I’m angry at myself.	PP	0.80
84.	I feel flat.	DPT	0.77
85.	I have to be the best in whatever I do.	SA	0.54
86.	I sacrifice pleasure, health, or happiness to meet my own standards.	DP	0.65
87.	I’m demanding of other people.	SA	0.57
88.	If I get angry, I can get so out of control that I injure other people.	EC	0.77
89.	I am invulnerable.	BA	0.04
90.	I’m a bad person.	PP	0.73
91.	I feel safe.	HC	0.84
92.	I feel listened to, understood, and validated.	HC	0.85
93.	It is impossible for me to control my impulses.	IC	0.71
94.	I destroy things when I’m angry.	EC	0.54
95.	By dominating other people, nothing can happen to you.	BA	0.59
96.	I act in a passive way, even when I don’t like the way things are.	CS	0.61
97.	My anger gets out of control.	EC	0.82
98.	I mock or bully other people.	BA	0.48
99.	I feel like lashing out or hurting someone for what he/she did to me.	AC	0.56
100.	I know that there is a “right” and a “wrong” way to do things; I try hard to do things the right way, or else I start criticizing myself.	DP	0.54
101.	I often feel alone in the world.	VC	0.85
102.	I feel weak and helpless.	VC	0.85
103.	I’m lazy.	UC	0.64
104.	I can put up with anything from people who are important to me.	CS	0.40
105.	I’ve been cheated or treated unfairly.	AC	0.55
106.	I feel left out or excluded.	VC	0.77
107.	I be little others.	BA	0.55
108.	I feel optimistic.	HC	0.83
109.	I feel I shouldn’t have to follow the same rules that other people do.	SA	0.25
110.	I’m pushing myself to be more responsible than most other people.	DP	0.34
111.	I can stand up for myself when I feel unfairly criticized, abused, or taken advantage of.	HA	0.58
112.	I don’t deserve sympathy when something bad happens to me.	PP	0.59
113.	I feel that nobody loves me.	VC	0.77
114.	I feel that I’m basically a good person.	HA	0.64
115.	When necessary, I complete boring and routine tasks in order to accomplish things I value.	HA	0.41
116.	I feel spontaneous and playful.	HC	0.25
117.	I can become so angry that I feel capable of killing someone.	EC	0.40
118.	I have a good sense of who I am and what I need to make myself happy.	HA	0.74

Note: VC = Vulnerable Child; AC = Angry Child; EC = Enraged Child; IC = Impulsive Child; UC = Undisciplined Child; HC = Happy Child; CS = Compliant Surrender; DPT = Detached Protector; DSS = Detached Self-Soother; SA = Self-Aggrandizer; BA = Bully and Attack; PP = Punitive Parent; DP = Demanding Parent; HA = Healthy Adult.

**Table 5:** Factor intercorrelations (Spearman’s ρ value with Bonferroni correction) between the Polish version of the Short SMI subscales (*n* = 640).

	VC	AC	EC	IC	UC	HC	CS	DPT	DSS	SA	BA	PP	DP
VC													
AC	0.57 **												
EC	0.37 **	0.59 **											
IC	0.42 **	0.58 **	0.71 **										
UC	0.48 **	0.40 **	0.32 **	0.47 **									
HC	−0.84 **	−0.47 **	−0.33 **	−0.34 **	−0.37 **								
CS	0.59 **	0.33 **	0.20 **	0.29 **	0.39 **	−0.47 **							
DPT	0.73 **	0.43 **	0.22 **	0.28 **	0.44 **	−0.70 **	0.48 **						
DSS	0.52 **	0.51 **	0.26 **	0.37 **	0.23 **	−0.46 **	0.36 **	0.44 **					
SA	0.10 *	0.49 **	0.34 **	0.34 **	0.19 **	−0.04	0.04	0.13 **	0.22 **				
BA	0.29 **	0.60 **	0.41 **	0.44 **	0.31 **	−0.26 **	0.21 **	0.32 **	0.30 **	0.54 **			
PP	0.73 **	0.49 **	0.41 **	0.44 **	0.39 **	−0.64 **	0.57 **	0.57 **	0.52 **	0.15 **	0.31 **		
DP	0.38 **	0.41 **	0.22 **	0.17 **	0.03	−0.34 **	0.34 **	0.31 **	0.50 **	0.39 **	0.27 **	0.52 **	
HA	−0.69 **	−0.32 **	−0.36 **	−0.43 **	−0.51 **	0.71 **	−0.51 **	−0.55 *	−0.34 **	0.03	−0.19 **	−0.67 **	−0.13 **

Note: ** *p* < 0.01; * *p* < 0.05; VC = Vulnerable Child; AC = Angry Child; EC = Enraged Child; IC = Impulsive Child; UC = Undisciplined Child; HC = Happy Child; CS = Compliant Surrender; DPT = Detached Protector; DSS = Detached Self-Soother; SA = Self-Aggrandizer; BA = Bully and Attack; PP = Punitive Parent; DP = Demanding Parent; HA = Healthy Adult.

**Table 6:** Means, standard deviations, and differences between the means of the Polish version of the Short SMI subscales and effect size in two subgroups (*n* = 640).

SMI Subscales	Clinical Group (*n* = 240)		Non-Clinical Group (*n* = 400)		δ	*t(df)*	*p*
	*m*	*SD*	*m*	*SD*			
Vulnerable Child	3.30	1.06	2.78	1.05	0.50	6.07(638)	<0.001
Angry Child	3.20	0.83	2.94	0.77	0.33	4.09(638)	<0.001
Enraged Child	1.81	0.69	1.68	0.63	0.21	2.54(638)	0.011
Impulsive Child	2.64	0.86	2.36	0.76	0.35	4.34(638)	<0.001
Undisciplined Child	3.43	0.97	3.17	0.93	0.28	3.38(638)	<0.001
Happy Child	2.98	0.95	3.53	0.98	−0.57	−6.97(638)	<0.001
Compliant Surrender	3.05	0.87	2.78	0.81	0.32	3.92(638)	<0.001
Detached Protector	2.80	0.98	2.46	0.83	0.38	4.48(439.38) *	<0.001
Detached Self-Soother	3.28	1.05	2.98	1.05	0.29	3.59(638)	<0.001
Self-Aggrandizer	2.80	0.75	2.81	0.79	−0.01	−0.14(638)	0.891
Bully and Attack	2.07	0.72	2.06	0.67	0.01	0.08(638)	0.934
Punitive Parent	2.49	0.98	2.11	0.75	0.46	5.22(405.57) *	<0.001
Demanding Parent	3.58	0.91	3.43	0.87	0.16	1.98(638)	0.048
Healthy Adult	3.81	0.85	4.15	0.78	−0.42	−5.17(638)	<0.001

Note: *m* = mean, *SD* = standard deviations; δ *=* Cohen’s δ clinical group versus non-clinical group; * the value of the *t*-statistic with the unequal variance correction applied.

**Table 7:** Correlation (Spearman’s ρ value with Bonferroni correction) between the Polish version of the Short SMI subscales and the *Temperament and Character Inventory subscales* (*n* = 311).

	VC	AC	EC	IC	UC	HC	CS	DPT	DSS	SA	BA	PP	DP	HA
TCI														
Exploratory excitability	−0.32 **	−0.10	−0.05	−0.01	−0.24 **	0.29 **	−0.34 **	−0.34 **	−0.07	0.20 **	0.03	−0.19 **	−0.11 *	0.28 **
Impulsiveness	0.06	0.17 **	0.18 **	0.31 **	0.21 **	−0.06	−0.01	0.03	0.08	0.04	0.10	0.06	−0.17 **	−0.14 *
Fear of uncertainty	0.36 **	0.17 **	0.12 *	0.09	0.25 **	−0.31 **	0.31 **	0.19 **	0.16 **	−0.15 **	0.01	0.24 **	0.08	−0.34 **
Attachment	−0.38 **	−0.19 **	−0.07	−0.09	−0.24 **	0.44 **	−0.21 **	−0.59 **	−0.16 **	0.01	−0.15 *	−0.32 **	−0.11	0.37 **
Dependence	0.21 **	−0.06	−0.03	−0.01	0.12 *	−0.14 *	0.45 **	−0.01	0.12 *	−0.14 *	−0.05	0.23 **	0.13 *	−0.29
Persistence	−0.09	0.01	−0.10	−0.16 **	−0.45 **	0.07	−0.11	−0.10	0.12 *	0.28 **	0.01	−0.03	0.42 **	0.23
Responsibility	−0.56 **	−0.44 **	−0.38 **	−0.44 **	−0.39 **	0.51 **	−0.56 **	−0.47 **	−0.38 **	−0.06	−0.22 **	−0.49 **	−0.21 **	0.53 **
Purposeful	−0.65 **	−0.33 **	−0.28 **	−0.35 **	−0.56 **	0.61 **	−0.54 **	−0.56 **	−0.34 **	0.05	−0.14 *	−0.53 **	−0.11	0.66 **
Resourcefulness	−0.53 **	−0.23 **	−0.24 **	−0.30 **	−0.54 **	0.50 **	−0.57 **	−0.41 **	−0.24 **	0.08	−0.06	−0.46 **	−0.05	0.65 **
Self-acceptance	−0.29 **	−0.50 **	−0.32 **	−0.33 **	−0.37 **	0.28 **	−0.16 **	−0.32 **	−0.31 **	−0.49 **	−0.42 **	−0.28 **	−0.27 **	0.24 **
Enlightened second nature	−0.60 **	−0.40 **	−0.33 **	−0.45 **	−0.69 **	0.54 **	−0.52 **	−0.50 **	−0.32 **	−0.06	−0.20 **	−0.54 **	−0.10	0.64 **
Social acceptance	−0.29 **	−0.45 **	−0.37 **	−0.34 **	−0.30 **	0.28 **	−0.17 **	−0.39 **	−0.23 **	−0.34 **	−0.38 **	−0.23 **	−0.21 **	0.24 **
Empathy	−0.34 **	−0.29 **	−0.27 **	−0.29 **	−0.36 **	0.37 **	−0.22 **	−0.44 **	−0.23 **	−0.17 **	−0.21 **	−0.28 **	−0.10	0.34 **
Helpfulness	−0.24 **	−0.35 **	−0.24 **	−0.18 **	−0.23 **	0.29 **	−0.04	−0.37 **	−0.18 **	−0.21 **	−0.29 **	−0.12 *	−0.08	0.19 **
Compassion	−0.14 *	−0.48 **	−0.38 **	−0.28 **	−0.29 **	0.16 **	0.02	−0.22 **	−0.09	−0.33 **	−0.43 **	−0.17 **	−0.07	0.14 *
Pure-hearted conscience	−0.10	−0.27 **	−0.18 **	−0.18 **	−0.18 **	0.12 *	0.01	−0.25 **	−0.08	−0.21 **	−0.27 **	−0.08	0.02	0.08

Note: TCI = Temperament and Character Inventory; VC = Vulnerable Child; AC = Angry Child; EC = Enraged Child; IC = Impulsive Child; UC = Undisciplined Child; HC = Happy Child; CS = Compliant Surrender; DPT = Detached Protector; DSS = Detached Self-Soother; SA = Self-Aggrandizer; BA = Bully and Attack; PP = Punitive Parent; DP = Demanding Parent; HA = Healthy Adult; ** *p* < 0.01; * *p* < 0.05; underlined figures reflect predicted associations.

**Table 8:** Correlation (Spearman’s ρ value with Bonferroni correction) between the Polish version of the Short SMI subscales and other theoretically linked constructs (*n* = 329).

	VC	AC	EC	IC	UC	HC	CS	DPT	DSS	SA	BA	PP	DP	HA
CISS														
*Task-oriented style*	−0.38 **	−0.09	−0.20 **	−0.27 **	−0.41 **	0.37 **	−0.28 **	−0.28 **	−0.08	0.16 **	−0.02	−0.35 **	0.12 *	0.65 **
*Emotion-oriented style*	0.74 **	0.52 **	0.41 **	0.46 **	0.46 **	−0.57 **	0.54 **	0.49 **	0.54 **	0.23 **	0.32 **	0.71 **	0.55 **	−0.57 **
*Avoidance-oriented style*	−0.03	0.16 **	0.13 *	0.21 **	0.23 **	0.13 *	0.02	−0.15 **	0.15 **	0.17 **	0.18 **	−0.01	−0.02	−0.02
*distraction*	0.29 **	0.30 **	0.26 **	0.35 **	0.42 **	−0.22 **	0.23 **	0.19 **	0.32 **	0.19 **	0.29 **	0.27 **	0.09	−0.30 **
*social diversion*	−0.39 **	−0.10	−0.07	−0.05	−0.08	0.46 **	−0.29	−0.50 **	−0.16 **	−0.03	−0.07	−0.31 **	−0.13 *	0.36 **
SES	−0.80 **	−0.42 **	−0.32 **	−0.39 **	−0.42 **	0.74 **	−0.55	−0.54 **	−0.47 **	−0.03	−0.23 **	−0.78 **	−0.43 **	0.74 **
STAXI-2														
*Anger-as-state*	0.32 **	0.33 **	0.45 **	0.36 **	0.20 **	−0.34 **	0.18	0.21 **	0.24 **	0.22 **	0.28 **	0.31 **	0.16 **	−0.33 **
*Anger-as-trait*	0.35 **	0.72 **	0.69 **	0.69 **	0.38 **	−0.28 **	0.17	0.18 **	0.37 **	0.55 **	0.49 **	0.32 **	0.31 **	−0.26 **
*Anger expression-out scale*	0.23 **	0.50 **	0.63 **	0.52 **	0.25 **	−0.14 *	−0.01	0.04	0.17 **	0.37 **	0.32 **	0.23 **	0.19 **	−0.14 **
*Anger expression-in scale*	0.43 **	0.29 **	−0.01	0.12 *	0.33 **	−0.34	0.49	0.52 **	0.45 **	0.23 **	0.26 **	0.40 **	0.38 **	−0.27 **
*Anger control-out scale*	−0.18 **	−0.41 **	−0.69 **	−0.55 **	−0.19 **	0.18 **	0.03	0.04	−0.11 *	−0.21 **	−0.22 **	−0.19 **	−0.10	0.29 **
*Anger control-in scale*	−0.23 **	−0.38 **	−0.60 **	−0.49 **	−0.21 **	0.24 **	−0.05	−0.06	−0.06	−0.26 **	−0.24 **	−0.23 **	−0.09	0.34 **

Note: CISS = *Coping Inventory for Stressful Situations*; SES = *Rosenberg Self-Esteem Scale*; STAXI-2 = *State–Trait Anger Expression Inventory-2*; VC = Vulnerable Child; AC = Angry Child; EC = Enraged Child; IC = Impulsive Child; UC = Undisciplined Child; HC = Happy Child; CS = Compliant Surrender; DPT = Detached Protector; DSS = Detached Self-Soother; SA = Self-Aggrandizer; BA = Bully and Attack; PP = Punitive Parent; DP = Demanding Parent; HA = Healthy Adult; ** *p* < 0.01; * *p* < 0.05; underlined figures reflect predicted associations.

## Data Availability

The data presented in this study are available on request from the corresponding author.

## Appendix A

**Table A1:** Description of 14 schema modes divided into four categories (based on Lobbesteal et al. [12]).

Category	Schema Modes	Description
Maladaptive Child Modes	Vulnerable Child	The individual experiences a sense of unhappiness, anxiety, sadness, and helplessness.
	Angry Child	The person experiences intense anger and even rage and feels frustrated and impatient when their needs go unmet.
	Enraged Child	The person experiences excessive anger, leading to out-of-control outbursts of aggression in which he or she may hurt others or destroy objects.
	Impulsive Child	A person acts on impulse or desires. He does not regard the consequences of his behavior and has difficulties deferring gratification.
	Undisciplined Child	The individual cannot push himself to complete routine, repetitive tasks, as he quickly gets frustrated and gives up.
Dysfunctional Coping Modes	Compliant Surrender	The individual is passive, submissive, requires reassurance and guarantees, and diminishes his value because of fear of conflict or rejection.
	Detached Protector	The person escapes the mental pain of unsatisfied needs by turning off all emotions, breaking ties with others, and rejecting anyone’s help. He behaves like a robot.
	Detached Self-Soother	The person avoids experiencing emotions by engaging in activities that soothe, stimulate, or distract him or her (such as workaholism, gambling, extreme sports, casual sex, or using drugs).
	Self-Aggrandizer	The individual tends toward competition and power, behaves pretentiously, downplays and uses others to get what he wants. He shows superiority and demands special treatment.
	Bully and Attack	The individual uses threats, bullying, and aggression to get what he wants or to protect himself from perceived harm.
Dysfunctional Parent Modes	Punitive Parent	This is the internalized voice of significant others who criticize or punish the individual. It results in self-hate, self-denial, self-harm, suicidal fantasies, and self-destructive behavior.
	Demanding Parent	This is a voice that pressures and pushes the individual to meet excessive standards. It expects perfectionism, maintaining order and tidiness, pursuing a high status, high productivity, and not wasting time.
Healthy Modes	Healthy Adult	He performs functions specific to adults, such as working, raising children, and taking responsibility. He also undertakes activities that are a pleasure, such as sex, pursues intellectual, aesthetic, and cultural interests, and takes care of his health and plays sports.
	Happy Child	The individual experiences inner peace because her basic emotional needs are satisfied. They feel loved, fulfilled, competent, secure, praised, valuable, understood, resilient, optimistic, and spontaneous. They feel connected to and cared for by others. Meanwhile, they have a sense of autonomy and control.
